# Factors Associated with Clinically Meaningful Pain Reduction Following Phase-I Complex Decongestive Therapy in Breast Cancer-Related Lymphedema

**DOI:** 10.3390/medsci14030415

**Published:** 2026-07-22

**Authors:** Lucia Demjanovič Kendrová, Wioletta Mikuľáková, Jakub Čuj, Pavol Nechvátal, Katarína Hnatová, Miloslav Gajdoš, Štefánia Andraščíková, Peter Takáč

**Affiliations:** 1 Department of Physiotherapy, Faculty of Health Care, University of Prešov, Partizánska 1, 08001 Prešov, Slovakia; wioletta.mikulakova@unipo.sk (W.M.); jakub.cuj@unipo.sk (J.Č.); pavol.nechvatal@unipo.sk (P.N.); katarina.hnatova@unipo.sk (K.H.); miloslav.gajdos@unipo.sk (M.G.); 2 Department of Midwifery, Faculty of Health Care, University of Prešov, Partizánska 1, 08001 Prešov, Slovakia; stefania.andrascikova@unipo.sk; 3 Department of Physical Medicine, Balneology and Medical Rehabilitation, Medical Faculty of P. J. Šafárik University, Rastislavova 43, 04001 Košice, Slovakia; peter.takac@upjs.sk; 4 Department of Physical and Rehabilitation Medicine, L. Pasteur University Hospital in Košice, Rastislavova 43, 04190 Košice, Slovakia

**Keywords:** lymphedema, breast cancer, complex decongestive therapy, pain, VAS, LYMQOL

## Abstract

**Background**: Upper-limb lymphedema after breast cancer treatment is associated with pain, functional limitations, and impaired quality of life. Although complex decongestive therapy (CDT) is standard conservative care, prospective evidence regarding factors associated with clinically meaningful response remains limited. We evaluated the short-term outcomes following Phase-I CDT and identified factors associated with clinically meaningful pain reduction. **Methods**: A prospective observational study was conducted in 94 women with breast cancer-related lymphedema undergoing a standardized 14-day Phase-I CDT program. Outcomes included limb circumference, pain intensity measured using the Visual Analogue Scale (VAS), and quality of life assessed with LYMQOL-Arm. Clinically meaningful improvement was defined a priori as a reduction of at least 2 points on the VAS (ΔVAS ≥ 2). Analyses included paired *t*-tests, Cohen’s *d*, multivariable logistic regression, analysis of covariance (ANCOVA), and receiver operating characteristic (ROC) analysis. **Results**: Significant reductions in limb circumference were observed across all measurement levels (3.08–5.83%; all *p* < 0.001). Pain intensity decreased from 5.53 ± 2.15 to 2.82 ± 1.41, with a mean reduction of 2.71 points (95% CI 2.32–3.11; *p* < 0.001) and a very large effect size (Cohen’s *d* = 1.40). All LYMQOL domains improved significantly. Higher baseline pain intensity was associated with a greater likelihood of achieving the predefined criterion for clinically meaningful improvement (OR 3.03; 95% CI 1.91–4.80), while older age was associated with reduced odds of response (OR 0.90; 95% CI 0.85–0.96). Baseline pain intensity demonstrated good discriminative performance (AUC 0.85). Circumference changes were not correlated with subjective improvement. **Conclusions**: Following the 14-day Phase-I CDT program, statistically significant reductions in total limb circumference, clinically meaningful pain reduction, and significant improvements in quality of life were observed. Exploratory analyses demonstrated an association between baseline pain intensity and the predefined responder outcome; however, this association is structurally influenced by the mathematical relationship between baseline VAS and the responder definition, baseline-dependent opportunity for improvement, and regression to the mean. Therefore, it should not be interpreted as evidence of an independent predictive effect and requires external validation before being considered for patient stratification or clinical decision-making. Because of the observational pre–post design without a control group, the observed changes cannot be attributed specifically to Phase-I CDT.

## 1. Introduction

As survival after breast cancer continues to improve, long-term treatment-related complications such as breast cancer-related lymphedema (BCRL) are becoming increasingly important [1,2]. Upper-limb lymphedema is a common complication of certain breast cancer treatments, particularly axillary lymph node dissection and radiotherapy, which disrupt normal lymphatic drainage pathways [3,4,5,6]. Although not all breast cancer treatments carry the same risk, BCRL remains one of the most burdensome long-term treatment sequelae, with a reported prevalence of approximately 20–30% depending on diagnostic criteria and follow-up duration [3,4]. Beyond limb swelling, BCRL is associated with pain, functional impairment, reduced upper-limb mobility, psychological distress, diminished quality of life, and socioeconomic consequences, including reduced work capacity and the need for long-term management [3,4]. The pathophysiology of BCRL involves chronic accumulation of protein-rich interstitial fluid resulting from impaired lymphatic transport, accompanied by persistent inflammation, adipose tissue remodeling, and progressive tissue fibrosis [5,7]. Although BCRL is considered a chronic condition, its symptoms can be effectively managed through appropriate rehabilitation interventions. Complex decongestive therapy (CDT) is widely regarded as the standard conservative treatment for BCRL [8,9,10]. CDT is a multimodal intervention consisting of manual lymphatic drainage, compression therapy, therapeutic exercise, skin care, and patient education. The physiological rationale for CDT is based on enhancing lymphatic transport, reducing interstitial fluid accumulation, improving the muscle pump mechanism, preventing tissue fibrosis, and supporting long-term self-management [8,9,10]. Previous studies have demonstrated that CDT reduces limb circumference and improves patient-reported outcome measures (PROMs); however, substantial heterogeneity remains regarding treatment protocols, outcome definitions, and follow-up duration [11,12,13]. Most published studies have primarily evaluated objective indicators of lymphedema, particularly changes in limb size or limb volume, whereas fewer investigations have simultaneously assessed objective changes together with patient-reported outcomes during intensive Phase-I CDT.

Therefore, this prospective study aimed to evaluate the effects of Phase-I CDT on limb circumference, pain intensity, functional status, and quality of life in women with BCRL, and to identify factors associated with clinically meaningful pain improvement.

## 2. Materials and Methods

### 2.1. Study Design and Participants

This prospective observational study included 94 women with breast cancer-related unilateral upper-limb lymphedema (BCRL) recruited consecutively between 10 November 2024 and 1 December 2025 from three rehabilitation centres in eastern Slovakia. All participants had previously undergone surgical and/or adjuvant breast cancer treatment, including axillary lymph node dissection, sentinel lymph node biopsy, and/or radiotherapy. This study was reported in accordance with the Strengthening the Reporting of Observational Studies in Epidemiology (STROBE) guidelines. The study was approved by the Ethics Committee of the Faculty of Health Care at the University of Prešov in Prešov (No. 06/2024; 4 November 2024), and all participants provided written informed consent. The research complied with the Declaration of Helsinki and national regulations.

Inclusion criteria were women with a history of breast cancer treatment and clinically confirmed unilateral upper-limb lymphedema, stable oncologic status, and the ability to complete the 14-day CDT program. Exclusion criteria were active infection, acute thrombosis, decompensated cardiovascular disease, advanced metastatic disease, non-adherence to treatment, or incomplete baseline or post-treatment assessments.

### 2.2. Intervention

The Phase-I CDT program consisted of daily manual lymphatic drainage (approximately 45 min) performed according to the Bechyně technique, multilayer short-stretch compression bandaging using Mobiderm^®^ foam padding (Thuasne, Levallois-Perret, France), skin care education, and supervised therapeutic exercises focused on lymphatic activation and upper-limb mobility (20–30 min per session). Exercise sessions were performed while wearing compression bandages and included vascular pump exercises, active range-of-motion exercises, and breathing exercises. Participants were hospitalized throughout the intervention period and completed supervised exercise sessions twice daily. All participants completed the full 14-day Phase-I CDT program. Treatment was delivered by certified physiotherapists with formal certification in manual lymphatic drainage and complete decongestive therapy (CDT), who routinely provided lymphedema management in specialised rehabilitation departments. All participating physiotherapists followed the same standardized Phase-I CDT protocol. Multilayer short-stretch compression bandaging with Mobiderm^®^ foam padding was standardized across all three participating centres. Compression pressure, bandage tension, and the number of compression layers were standardized according to the protocol used at each centre; however, these parameters were not instrumentally measured. Formal treatment fidelity monitoring was not performed. The 14-day duration reflects the standard inpatient Phase-I CDT protocol routinely used in the participating clinical centres and corresponds to the intensive treatment phase commonly applied in rehabilitation practice in Slovakia. The study was not designed or powered to evaluate centre-specific differences, and centre-related variability was therefore not formally assessed.

### 2.3. Outcome Measures

#### 2.3.1. Limb Circumference

Lymphedema severity was evaluated using circumferential measurements of the affected upper limb, a clinically accessible and reliable method with high intra- and inter-rater consistency [14]. Measurements were performed according to a standardized protocol at predefined anatomical landmarks by the same trained physiotherapist at each assessment, thereby minimizing measurement variability. The sum of circumference measurements was calculated as the sum of all measured anatomical segments. This composite measure was used for descriptive purposes to summarize circumferential changes across the upper limb and was not intended to represent true limb volume. Assessments were performed at two time points: baseline (Day 0, before initiation of CDT) and immediately after completion of the 14-day intervention (Day 14). Standardized anatomical levels included the dorsum of the hand, wrist, maximal forearm circumference, elbow, and 5 cm, 10 cm, and 15 cm above the elbow, as well as the axillary region.

#### 2.3.2. Quality of Life (QoL)

Quality of life was assessed using the LYMQOL-Arm questionnaire, which has demonstrated strong internal consistency and responsiveness to clinical change in populations with upper-limb lymphedema [15]. The instrument evaluates four domains: function, appearance, symptoms, and mood, and includes a single overall QoL score. Items within each domain are rated on a four-point Likert scale (1 = not at all; 4 = a lot). The global quality-of-life score is rated on a 0–10 scale, where higher scores reflect better perceived QoL. The LYMQOL-Arm has shown good reliability and sensitivity to change [15].

#### 2.3.3. Pain Assessment

Pain intensity was assessed using the Visual Analogue Scale (VAS) ranging from 0 (no pain) to 10 (worst imaginable pain). The VAS is a validated and reliable measure of subjective pain and is sensitive to clinically meaningful change [16].

The minimal clinically important difference (MCID) for VAS is typically reported as 1–2 points on the 0–10 scale, though values vary with baseline severity and methodological approaches [7,17,18]. Because no BCRL-specific MCID has been established for pain measured with the VAS, the threshold of ΔVAS ≥ 2 was selected pragmatically based on the general pain literature and studies involving upper extremity conditions. For upper extremity conditions, MCID estimates of ~1.6–1.9 points have been described, suggesting the use of ΔVAS ≥ 2 as a conservative threshold for clinically meaningful improvement [19]. Therefore, a reduction of at least 2 points (ΔVAS ≥ 2) was considered a clinically meaningful improvement from the patient’s perspective and was used to classify participants as responders.

#### 2.3.4. Study Endpoints

The primary endpoint was clinically meaningful pain improvement, defined a priori as a reduction of at least 2 points on the VAS (ΔVAS ≥ 2).

Secondary endpoints included absolute change in pain intensity, changes in limb circumference, changes in LYMQOL-Arm scores, and identification of factors associated with treatment response.

#### 2.3.5. Sample Size Justification

The sample size (*n* = 94) was sufficient to detect a large pre–post effect in VAS pain (Cohen’s d ≥ 0.80) with 90% power. For the binary outcome (ΔVAS ≥ 2), the sample size allowed exploratory multivariable logistic regression analyses. However, given the relatively small number of non-responders, the regression results should be interpreted with appropriate caution.

### 2.4. Statistical Analysis

Statistical analyses were performed using SPSS 30 and Statistica 14. Descriptive data are reported as mean ± SD. Normality was assessed using the Shapiro–Wilk test and visual inspection.

Pre–post changes were analysed using paired *t*-tests, with effect sizes expressed as Cohen’s d. Associations between changes (ΔVAS, ΔLYMQOL) were examined using Pearson correlation coefficients.

Factors associated with pain change (ΔVAS) were assessed using multiple linear regression, while clinically meaningful improvement (ΔVAS ≥ 2) was analysed using multivariable logistic regression, reporting odds ratios (ORs) and 95% confidence intervals (CIs). ANCOVA was performed to evaluate factors independently associated with post-treatment pain intensity after adjustment for baseline pain and relevant covariates. This analysis was intended to assess residual pain following treatment rather than the probability of achieving clinically meaningful improvement.

The discriminative ability of baseline pain intensity to identify participants achieving clinically meaningful pain improvement (ΔVAS ≥ 2) was evaluated using receiver operating characteristic (ROC) analysis. The optimal cut-off value was determined using Youden’s index. Model performance was summarized using R^2^ and adjusted R^2^ for linear regression and McFadden’s pseudo-R^2^ for logistic regression. All clinically relevant variables were entered simultaneously into the multivariable models to evaluate their independent associations with treatment outcomes. No missing data were present for any variables included in the analyses; therefore, no imputation procedures were required.

Statistical significance was set at *p* < 0.05, and effect sizes with 95% confidence intervals are reported where appropriate. No formal sensitivity analyses were performed because the multivariable analyses were considered exploratory and hypothesis-generating.

## 3. Results

**Figure 1:**
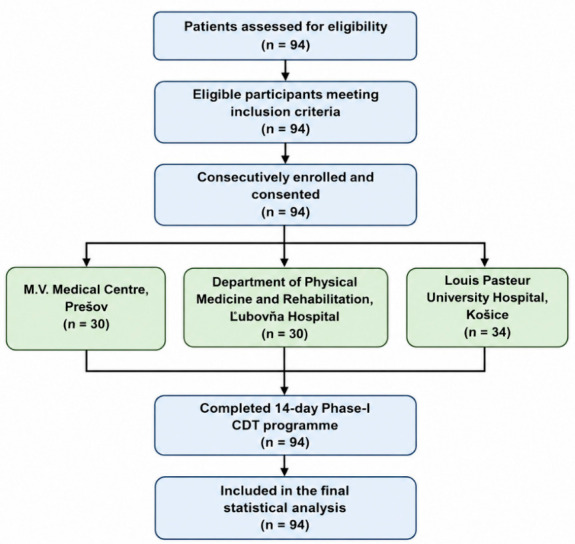
Flow diagram of participant recruitment and study inclusion. All consecutive patients meeting the eligibility criteria agreed to participate, completed the 14-day Phase-I complex decongestive therapy (CDT) programme, and were included in the final analyses.

A total of 94 consecutive women were recruited from three rehabilitation centres: M.V. Medical Centre, Prešov (*n* = 30), Department of Physical Medicine and Rehabilitation, Ľubovňa Hospital (*n* = 30), and Louis Pasteur University Hospital, Košice (*n* = 34). The participant flow is presented in Figure 1. Baseline demographic and clinical characteristics are summarized in Table 1. All participants completed the 14-day Phase-I CDT program and both pre- and post-treatment assessments.

Significant reductions were observed across all anatomical measurement levels (all *p* < 0.001). The sum of circumference measurements decreased by 4.21%, reflecting an overall reduction in circumferential measurements following Phase-I CDT. Effect sizes ranged from moderate to large (Cohen’s *d* = 0.38–0.81), indicating consistent reductions across all anatomical measurement sites (Table 2).

Pain intensity decreased significantly following Phase-I CDT, with a very large effect size (Cohen’s d = 1.40). Overall, 63 participants (67.0%) achieved the predefined criterion for clinically meaningful pain improvement (responders), whereas 31 (33.0%) did not (non-responders) (Table 3).

All LYMQOL domains improved significantly after treatment, with the greatest effect observed in the symptoms domain (Cohen’s d = 1.56). Improvements were also observed in function, appearance, mood, and overall quality of life (Table 4).

Reductions in pain were strongly correlated with improvements in global quality of life (r = 0.703, *p* < 0.001) and with several LYMQOL subdomains (r = 0.52–0.71). In contrast, changes in limb circumference showed no significant correlations with pain or QoL outcomes (all *p* > 0.05) (Table 5).

In the multivariable logistic regression model, higher baseline pain intensity was associated with a greater likelihood of achieving clinically meaningful improvement, whereas older age was associated with a lower likelihood of response. Baseline LYMQOL scores were not significantly associated with treatment response (Table 6).

ROC analysis explored the ability of baseline pain intensity to discriminate between participants who did and did not achieve the predefined criterion for clinically meaningful pain improvement. However, because baseline VAS is mathematically embedded in the definition of the responder outcome (ΔVAS ≥ 2), these findings should be interpreted with considerable caution. The exploratory optimal cut-off value was VAS ≥ 5, with a sensitivity of 0.89 and a specificity of 0.65. However, because specificity was only moderate, the proposed threshold should be considered exploratory, interpreted cautiously, and should not be used as a standalone clinical decision-making tool.

After adjustment for baseline VAS, age, and baseline symptom burden, only baseline pain remained significantly associated with post-treatment pain intensity. Although participants with higher baseline pain demonstrated larger absolute reductions in pain, they also tended to report higher residual pain following treatment. Thus, greater absolute improvement did not necessarily correspond to lower post-treatment pain intensity (Table 7).

## 4. Discussion

Statistically significant reductions in total limb circumference and improvements in quality of life were observed following the 14-day Phase-I CDT program. In addition, clinically meaningful pain reduction was achieved in 67.0% of participants. Pain reduction was substantial (Cohen’s d = 1.40; mean ΔVAS = 2.71 points), consistent with favourable short-term changes following intensive Phase-I CDT reported in previous studies [20].

These findings align with previous prospective and interventional studies showing meaningful edema reduction and symptom improvement after intensive CDT. Four-week post-mastectomy protocols similarly improved circumference, function, and pain [21], and retrospective analyses after 15–30 sessions reported consistent volume decreases [19]. Randomized comparisons of therapist-led and self-administered CDT demonstrated sustained symptom reduction [22]. CDT has also yielded clinically relevant relief in palliative settings [23], and functional gains such as improved balance and gait are well documented [24]. Systematic reviews consistently reaffirm CDT as the gold standard conservative treatment for BCRL with measurable QoL benefits [7,25]. Although statistically significant improvements were observed across all LYMQOL-Arm domains, no universally accepted minimal clinically important difference (MCID) has yet been established for this questionnaire. Therefore, the clinical significance of these changes cannot currently be interpreted using a validated threshold.

A key observation is the lack of correlation between circumference reduction and improvements in pain or QoL, consistent with evidence showing that functional gains may occur independently of volume change, especially when CDT is initiated early or baseline excess is lower [22]. These findings reinforce the need for a multidimensional assessment of treatment outcomes, as objective limb measurements alone may underestimate changes in patient-reported outcomes following Phase-I CDT, particularly given the substantial functional and psychosocial impact of BCRL [3,4]. However, this finding should be interpreted cautiously. Circumferential measurements are widely used in clinical practice because of their accessibility, low cost, feasibility, and good reliability when performed according to standardized protocols [14]. However, they provide an indirect estimate of overall limb size rather than true limb volume and may be less sensitive than volumetric assessment, perometry, or bioimpedance spectroscopy for detecting subtle changes in tissue composition and fluid distribution [8,14]. Therefore, the absence of correlation between structural and subjective outcomes may partly reflect limitations in measurement sensitivity rather than a true dissociation between physical and symptomatic improvement.

Responder analysis showed that baseline pain intensity was the variable most strongly associated with the predefined criterion of clinically meaningful improvement (OR ≈ 3; AUC 0.85), whereas older age was associated with a lower probability of treatment response. This aligns with literature highlighting the role of tissue factors (fibrosis, adipose remodeling), disease stage, and early treatment initiation in shaping CDT outcomes [17,26]. An additional contribution of the present study is the application of ROC analysis together with multivariable regression methods to explore associations between baseline symptom burden and treatment response [27,28].

The complementary ANCOVA analysis provided additional insight by demonstrating that higher baseline pain remained associated with higher post-treatment pain after adjustment for relevant covariates. Thus, although participants with higher baseline pain experienced larger absolute reductions in pain, they also tended to report greater residual pain following treatment. These findings indicate that larger absolute improvement should not be interpreted as a better overall clinical outcome.

Our definition of clinically meaningful improvement (ΔVAS ≥ 2) aligns with empirical MCID estimates: typically, 1–2 points on the 0–10 scale, varying with baseline severity and methodological factors [15,18,29,30], and ~1.6–1.9 points in upper extremity populations [31]. An additional methodological consideration relates to the potential influence of regression to the mean and mathematical coupling between baseline values and change scores. Patients with higher baseline pain levels inherently have greater opportunity to achieve a clinically meaningful reduction in pain, which may partly contribute to the observed association between baseline VAS and the predefined responder outcome. In addition, because baseline VAS is mathematically embedded in the definition of the responder outcome (ΔVAS ≥ 2), the observed association between baseline pain intensity and achievement of the predefined responder criterion is inherently influenced by mathematical coupling, baseline-dependent opportunity for improvement, and regression to the mean. Consequently, the observed odds ratios and the ROC-derived threshold should not be interpreted as evidence that baseline pain independently predicts treatment responsiveness. Rather, these findings should be regarded as exploratory and hypothesis-generating and should not be used for patient stratification, treatment prioritisation, or clinical decision-making without external validation.

Baseline pain assessment may facilitate patient counseling by helping to establish realistic expectations regarding short-term symptom improvement and may contribute to personalized treatment planning. These observations align with current recommendations emphasizing personalized treatment protocols, routine use of patient-reported outcome measures (PROMs), and patient education and self-management [25]. Previous studies have consistently reported improvements in function and quality of life following CDT, supporting the clinical relevance of the present findings [7]. However, this observation should be interpreted cautiously. Although the ROC analysis demonstrated high sensitivity (0.89), the specificity was only moderate (0.65), indicating that a proportion of patients may be incorrectly classified as likely responders (false positives). Consequently, the proposed VAS ≥ 5 threshold should not be interpreted as a standalone clinical decision-making tool but rather as an exploratory finding requiring external validation before any potential clinical application.

Several mechanisms may explain the observed association between higher baseline pain intensity and a greater likelihood of clinically meaningful pain improvement following Phase-I CDT. Higher pain levels may reflect increased tissue tension associated with lymphatic fluid accumulation, persistent inflammation, and progressive tissue changes, including fibrosis, which are recognized components of BCRL pathophysiology [5]. Patients with greater symptom burden may also have more opportunity for measurable improvement during intensive CDT. However, baseline pain may also act as a surrogate marker for important clinical characteristics that were not systematically assessed in the present study, including body mass index, type of breast surgery, extent of axillary lymph node dissection, radiotherapy characteristics, ongoing systemic therapies, lymphedema stage, disease chronicity, fibrosis severity, and duration of symptoms. Consequently, the observed associations may partly reflect residual confounding rather than independent effects of baseline pain intensity. The absence of these variables may also have influenced the stability of the multivariable regression models and may limit the generalizability of the findings. Therefore, the results should be interpreted cautiously and confirmed in future studies incorporating more comprehensive clinical characterization and external validation [5,25].

Finally, this study is among the few prospective Phase-I CDT analyses to systematically examine factors associated with subjective clinical response while integrating objective outcomes, patient-reported outcome measures, and multivariable statistical analyses. These findings contribute to a better understanding of the relationship between baseline symptom burden and short-term treatment response and may help inform future research aimed at developing more personalized rehabilitation strategies.

### 4.1. Strengths and Limitations

Strengths of this study include its prospective multicentre design, the use of a standardized Phase-I CDT protocol, and the combined assessment of objective outcomes and validated patient-reported outcome measures (PROMs). Furthermore, the application of multivariable statistical modelling enabled exploratory analyses of factors associated with clinically meaningful treatment response.

Limitations include: (i) The absence of a control group, limiting causal inference; therefore, natural fluctuations, hospitalization, attention effects, regression to the mean, and concomitant symptomatic management may have contributed to the observed improvements. (ii) There was a lack of medium- and long-term follow-up assessments, including evaluations at 3 and 6 months after completion of treatment. Because outcomes were assessed only immediately after completion of the 14-day Phase-I CDT program, the present findings relate exclusively to short-term post-treatment effects and do not permit conclusions regarding the long-term durability or sustainability of the observed improvements. (iii) There were several potentially important clinical variables, including body mass index (BMI), type of breast surgery, extent of axillary lymph node dissection, radiotherapy characteristics, systemic oncological treatments, medication use, comorbidities, employment status, social factors, lymphedema stage, disease chronicity, fibrosis severity, and duration of symptoms, were not systematically collected and therefore could not be included in the multivariable analyses; consequently, residual confounding cannot be excluded, and the stability and generalizability of the exploratory regression models may be limited. (iv) There was possible variability in bandaging intensity and manual therapy techniques. (v) There was the potential for regression to the mean and mathematical coupling between baseline values and change scores. (vi) No formal sensitivity analyses (e.g., bootstrap validation or penalised regression) were performed because the multivariable analyses were considered exploratory and hypothesis-generating. Consequently, the robustness and stability of the multivariable models should be interpreted with appropriate caution, particularly given the relatively small number of non-responders. These limitations should be considered when interpreting the findings. Future multicenter randomized studies incorporating longer follow-up periods, more comprehensive patient characterization, objective tissue assessment methods (e.g., elastography), and external validation are warranted to further validate and extend the present findings.

### 4.2. Future Research Directions

Future studies should: (i) validate the observed associations and the proposed baseline VAS threshold in independent cohorts; (ii) examine how symptom burden interacts with tissue characteristics such as fibrosis and adipose remodeling to refine personalized CDT protocols; (iii) compare specific CDT components and dosing in pragmatic randomized trials; (iv) apply standardized MID/MCID procedures for PROMs in line with current methodological recommendations [32]; (v) incorporate long-term follow-up to evaluate both the durability of clinical outcomes and the oncological safety of CDT, including systematic monitoring for local recurrence and metastatic disease; and (vi) investigate the contribution of structured home exercise programs and self-management strategies, including adherence monitoring, to the long-term effectiveness of CDT.

## 5. Conclusions

Statistically significant reductions in total limb circumference and improvements in quality of life were observed following the 14-day Phase-I CDT program in women with breast cancer-related lymphedema. Clinically meaningful pain reduction was achieved in the majority of participants. The lack of correlation between structural and subjective outcomes highlights the importance of evaluating treatment effects beyond changes in limb circumference alone. Exploratory analyses demonstrated an association between baseline pain intensity and achievement of the predefined criterion for clinically meaningful pain improvement. However, this association is structurally influenced by the mathematical relationship between baseline VAS and the responder definition and may also partly reflect regression to the mean. Therefore, it should not be interpreted as evidence of an independent predictive effect. These findings further suggest that greater absolute pain reduction does not necessarily correspond to lower residual pain following treatment. Consequently, these findings should not be used for patient selection or treatment prioritisation without external validation. Because of the observational pre–post design and the absence of a control group, the observed changes cannot be attributed specifically to Phase-I CDT. These findings support comprehensive outcome assessment and may help inform future research and individualized lymphedema management.

## Figures and Tables

**Table 1:** Baseline demographic and clinical characteristics of the study participants (*N* = 94).

**Study Population**	
Number of participants	94
Recruitment period	10 November 2024–1 December 2025
**Participating centres**	
M. V. Medical Centre, Prešov	*n* = 30
Ľubovňa Hospital	*n* = 30
Louis Pasteur University Hospital, Košice	*n* = 34
**Demographic characteristics**	
Age (years), mean ± SD	58.55 ± 11.48
**Baseline clinical characteristics**	
Pain intensity (VAS), mean ± SD	5.53 ± 2.15
Sum of limb circumferences (cm), mean ± SD	225.86 ± 17.40
**Baseline LYMQOL-Arm scores**	
Function	1.76 ± 0.43
Appearance	1.82 ± 0.46
Symptoms	2.11 ± 0.48
Mood	1.68 ± 0.54
Overall QoL	5.14 ± 1.81

Note: SD—standard deviation; VAS—Visual Analogue Scale; LYMQOL—Lymphedema Quality of Life Questionnaire.

**Table 2:** Limb Circumference Reduction.

Variable	Baseline (Mean ± SD)	Post-Treatment (Mean ± SD)	% Reduction	Mean Difference (95% CI)	t (93)	*p*	Cohen d
Dorsum of the hand	20.03 ± 1.54	19.35 ± 1.49	3.40%	0.68 (0.51–0.85)	7.87	<0.001	0.81
Wrist	18.12 ± 1.88	17.56 ± 1.76	3.08%	0.56 (0.32–0.79)	4.71	<0.001	0.49
Maximal forearm circumference	25.22 ± 3.66	24.14 ± 3.52	4.26%	1.08 (0.57–1.58)	4.24	<0.001	0.44
Elbow	28.65 ± 3.37	27.72 ± 3.11	3.27%	0.93 (0.43–1.44)	3.69	<0.001	0.38
5 cm above the elbow	30.39 ± 3.54	28.62 ± 2.15	5.83%	1.77 (0.96–2.58)	4.33	<0.001	0.45
10 cm above the elbow	32.39 ± 3.77	31.02 ± 3.62	4.22%	1.37 (0.86–1.88)	5.30	<0.001	0.55
15 cm above the elbow	33.64 ± 3.28	31.68 ± 2.13	5.83%	1.96 (1.26–2.67)	5.52	<0.001	0.57
Axilla	37.41 ± 4.05	36.24 ± 3.92	3.11%	1.17 (0.75–1.58)	5.63	<0.001	0.58
Sum of circumference measurements	225.86 ± 17.40	216.34 ± 14.53	4.21%	9.52 (6.82–12.21)	7.01	<0.001	0.72

**Table 3:** Changes in pain intensity following Phase-I CDT.

Variable	Baseline (Mean ± SD)	Post-Treatment (Mean ± SD)	Mean Difference (95% CI)	t (93)	*p*	Cohen d
Pain intensity (VAS)	5.53 ± 2.15	2.82 ± 1.41	2.71 (2.32–3.11)	13.61	<0.001	1.40

**Table 4:** Quality of Life Outcomes.

Variable	Baseline (Mean ± SD)	Post-Treatment (Mean ± SD)	Mean Difference (95% CI)	t (93)	*p*	Cohen d
Function	1.76 ± 0.43	1.52 ± 0.34	0.24 (0.19–0.30)	9.43	<0.001	0.97
Appearance	1.82 ± 0.46	1.53 ± 0.37	0.29 (0.23–0.35)	9.24	<0.001	0.95
Symptoms	2.11 ± 0.48	1.56 ± 0.43	0.55 (0.47–0.62)	15.09	<0.001	1.56
Mood	1.68 ± 0.54	1.32 ± 0.34	0.36 (0.27–0.45)	7.71	<0.001	0.80
Overall QoL	5.14 ± 1.81	7.20 ± 1.50	2.06 (1.64–2.49)	−9.58	<0.001	0.99

Note: Lower scores in the Function, Appearance, Symptoms, and Mood domains indicate better quality of life; therefore, mean differences were calculated as baseline minus post-treatment values. For the Overall QoL score, higher scores indicate better quality of life (10 = best possible QoL); therefore, mean differences were calculated as post-treatment minus baseline. Accordingly, positive mean differences indicate improvement across all outcomes.

**Table 5:** Pearson correlations between objective and patient-reported outcome changes.

Variable	ΔVAS	ΔQOL	ΔFunction	ΔAppearance	ΔSymptoms	ΔMood	ΔCircumference
ΔVAS	—	0.703 (*p* < 0.001)	0.539 (*p* < 0.001)	0.709 (*p* < 0.001)	0.384 (*p* < 0.001)	0.593 (*p* < 0.001)	0.040 (*p* = 0.700)
ΔQOL	0.703 (*p* < 0.001)	—	0.621 (*p* < 0.001)	0.682 (*p* < 0.001)	0.633 (*p* < 0.001)	0.690 (*p* < 0.001)	−0.023 (*p* = 0.827)
ΔFunction	0.539 (*p* < 0.001)	0.621 (*p* < 0.001)	—	0.670 (*p* < 0.001)	0.580 (*p* < 0.001)	0.522 (*p* < 0.001)	0.110 (*p* = 0.290)
ΔAppearance	0.709 (*p* < 0.001)	0.682 (*p* < 0.001)	0.670 (*p* < 0.001)	—	0.542 (*p* < 0.001)	0.637 (*p* < 0.001)	0.109 (*p* = 0.295)
ΔSymptoms	0.384 (*p* < 0.001)	0.633 (*p* < 0.001)	0.580 (*p* < 0.001)	0.542 (*p* < 0.001)	—	0.508 (*p* < 0.001)	0.007 (*p* = 0.943)
ΔMood	0.593 (*p* < 0.001)	0.690 (*p* < 0.001)	0.522 (*p* < 0.001)	0.637 (*p* < 0.001)	0.508 (*p* < 0.001)	—	0.038 (*p* = 0.717)
ΔCircumference	0.040 (*p* = 0.700)	−0.023 (*p* = 0.827)	0.110 (*p* = 0.290)	0.109 (*p* = 0.295)	0.007 (*p* = 0.943)	0.038 (*p* = 0.717)	—

**Table 6:** Multivariable logistic regression analysis of factors associated with clinically meaningful pain improvement (ΔVAS ≥ 2).

Variable	Odds Ratio (OR)	95% CI	*p*-Value
Intercept	8.94	0.17–470.25	0.279
Age (years)	0.90	0.85–0.96	0.001
Baseline pain intensity (VAS)	3.03	1.91–4.80	<0.001
Baseline symptom burden (LYMQOL)	0.65	0.19–2.29	0.505

Note. *n* = 94. ΔVAS defined as baseline minus post-treatment score; clinically meaningful improvement, defined a priori as a reduction of at least 2 VAS points (MCID threshold). Model fit: Pseudo-R^2^ = 0.443; LR χ^2^(3) = 52.77; *p* < 0.001. OR = odds ratio; CI = confidence interval.

**Table 7:** Adjusted ANCOVA Model Predicting Post-Treatment Pain Intensity (VAS).

Predictor	Unstandardized Coefficient (B)	Standard Error (SE)	95% CI	*p*-Value
Intercept	−0.111	0.891	−1.88–1.66	0.901
Baseline pain intensity (VAS)	0.287	0.063	0.16–0.41	<0.001
Age (years)	0.013	0.012	−0.01–0.04	0.277
Baseline symptom burden (LYMQOL)	0.285	0.273	−0.26–0.83	0.299

Note. *n* = 94. Dependent variable: post-treatment pain intensity (VAS). Model adjusted for baseline pain, age, and baseline symptom burden. Model fit: R^2^ = 0.245; Adjusted R^2^ = 0.220; F(3, 90) = 9.76; *p* < 0.001. B = unstandardized regression coefficient; SE = standard error; CI = confidence interval.

## Data Availability

The data presented in this study are available upon reasonable request from the corresponding author due to ethical and data protection restrictions involving patient information.

## Abbreviations

ANCOVA	Analysis of Covariance
AUC	Area Under the Curve
BCRL	Breast Cancer-Related Lymphedema
CDT	Complex Decongestive Therapy
CI	Confidence Interval
LYMQOL	Lymphedema Quality of Life Questionnaire
MCID	Minimal Clinically Important Difference
OR	Odds Ratio
QoL	Quality of Life
ROC	Receiver Operating Characteristic
SD	Standard Deviation
VAS	Visual Analogue Scale
